# Clinical and Genomic Factors Associated with Elacestrant Outcomes in *ESR1-*Mutant Metastatic Breast Cancer

**DOI:** 10.1158/1078-0432.CCR-25-3033

**Published:** 2025-11-07

**Authors:** Maxwell R. Lloyd, Caroline M. Weipert, Azka Ali, Sheila R. Solomon, Jayati Saha, Marla D. Lipsyc-Sharf, Erika P. Hamilton, Kevin Kalinsky, Adam M. Brufsky, Aditya Bardia, Nicole Zhang, Seth A. Wander

**Affiliations:** 1 Beth Israel Deaconess Medical Center, Boston, Massachusetts.; 2 Guardant Health, Inc., Redwood City, California.; 3 Cleveland Clinic Foundation, Taussig Cancer Institute, Cleveland, Ohio.; 4 UCLA Health Jonsson Comprehensive Cancer Center, Los Angeles, California.; 5 Sarah Cannon Research Institute, Nashville, Tennessee.; 6 Winship Cancer Institute at Emory University, Atlanta, Georgia.; 7 University of Pittsburgh Medical Center, Pittsburgh, Pennsylvania.; 8 Massachusetts General Hospital Cancer Center, Boston, Massachusetts.

## Abstract

**Purpose:**

*ESR1* mutations mediate resistance to antiestrogen therapy in hormone receptor–positive metastatic breast cancer (MBC). Elacestrant, an oral selective estrogen receptor degrader, improves progression-free survival over standard endocrine therapy in *ESR1*-mutant MBC. We assessed real-world elacestrant use and clinical–genomic factors associated with outcomes.

**Experimental Design:**

This study used the GuardantINFORM database, linking >42,000 real-world breast cancer cases with sequencing and claims data. We included patients with activating *ESR1* mutations detected <6 months before elacestrant initiation (January 2023–March 2024). Outcomes of time-to-treatment-discontinuation, time-to-next-treatment (TTNT), and overall survival were estimated with Kaplan–Meier and Cox regression analysis, adjusting for clinical variables.

**Results:**

We identified 756 patients (76% with prior cyclin-dependent kinase-4/6 inhibitor and 38% with prior chemotherapy exposure), and 742 (98.2%) were evaluable for outcomes. The median TTNT was 6.4 months, and the time-to-treatment-discontinuation was 4.6 months. In those with ≤1 prior lines of metastatic therapy, the TTNT was 8.8 months, compared with 6.0 months in the third-line setting. Prior fulvestrant exposure trended toward shorter treatment duration (hazard ratio, 1.19; 95% confidence interval, 0.91–1.56). Higher *ESR1* polyclonality (≥4 alterations; 11% of patients) correlated with a shorter TTNT of 5.2 months (hazard ratio, 1.44; 95% confidence interval, 1.01–2.06), but efficacy was consistent across *ESR1* alleles (e.g., *Y537S* and *D538G*). Disease with dual *ESR1* and PI3K pathway mutations (*PIK3CA*, *AKT1*, and *PTEN*) had a median TTNT of 5.2 months.

**Conclusions:**

In *ESR1*-mutant MBC, elacestrant treatment durations support the routine use of elacestrant monotherapy in appropriately selected patients. For patients with concurrent *ESR1* and PI3K pathway mutations, single-agent activity was comparable with outcomes observed in phase III studies.

*See related article by Rugo et al., p. 179*

**Translational Relevance**

In routine practice, there are limited data evaluating the efficacy of elacestrant for *ESR1*-mutant metastatic breast cancer and clinical–genomic factors associated with differences in therapeutic outcomes. In this real-world cohort of more than 750 patients with *ESR1-*mutant breast tumors, the median time-to-next-treatment on elacestrant was 6.4 months overall and 8.8 months in patients with one or fewer prior lines of metastatic therapy. Elacestrant was similarly effective across different *ESR1* allele variants, and a shorter treatment duration was observed in a small proportion of patients with higher *ESR1*-mutant polyclonality burden (≥4 concurrent alterations). Among patients with disease harboring dual *ESR1* and PI3K pathway mutations (a population with multiple approved targeted therapy options), the median time-to-next-treatment was found to be 5.2 months. Overall, elacestrant demonstrates encouraging real-world single-agent activity in patients with *ESR1*-mutant metastatic breast cancer, and biomarkers of response hold promise for refining its clinical use.

## Introduction

More than 250,000 people are newly diagnosed with breast cancer each year in the United States, and significant morbidity and mortality risks can arise from cancer-related complications and treatments (1). Hormone receptor–positive (HR+) metastatic breast cancer (MBC) is common and incurable, and improving therapeutic options is an important area of active research (2). Standard first-line treatment often involves an antiestrogen therapy combined with a cyclin-dependent kinase (CDK)4/6 inhibitor (3–5), however, the emergence of drug resistance in these tumors is inevitable.

Nearly 80% of breast cancers overexpress the estrogen receptor (ER), which is a ligand-dependent transcription factor that is activated by estrogen and promotes downstream tumor growth signaling (6). Oncogenic mutations in ER 1 (*ESR1*), the gene that encodes the ER, can promote constitutive estrogen-independent receptor signaling (7). Prior work has shown that activating *ESR1* mutations are infrequent in *de novo* MBC but arise as an important and common mechanism of acquired resistance under the selective pressure of aromatase inhibitor therapy, which exerts its antitumor effect by depleting circulating estrogen (8). Previous work also suggests that the *ESR1* allele-specific impact on drug sensitivity may vary and that *Y537S* may be associated with more aggressive biology and worse outcomes compared with other variants (9, 10).

Fulvestrant is a selective ER degrader (SERD) that seems, to varying degrees in different populations, to retain antitumor activity in *ESR1*-mutant MBC (11–14). Several next-generation orally bioavailable SERDs and novel antiestrogen therapies in development have shown promise for the management of *ESR1*-altered disease (15). The emergence of these next-generation endocrine therapies, both as monotherapy and in combination regimens, is part of a growing armamentarium of precision breast cancer therapeutics that have potential to improve patient outcomes (2).

Elacestrant is the first oral SERD that is FDA-approved for patients with *ESR1-*mutant HR+/HER2− MBC. The phase III EMERALD trial demonstrated that elacestrant yielded a statistically significant improvement in progression-free survival (PFS) compared with standard-of-care endocrine therapy [hazard ratio (HR), 0.70; 95% confidence interval (CI), 0.55–0.88]. In this trial, patients were required to have prior CDK4/6 inhibitor treatment, and prior fulvestrant and/or chemotherapy was allowed. The magnitude of improvement in PFS on elacestrant was greatest in the *ESR1*-mutant population (HR, 0.55; 95% CI, 0.39–0.77), and PFS was not significantly improved with elacestrant in *ESR1* wild-type patients (HR, 0.86; 95% CI, 0.63–1.19), suggesting that the PFS benefit seen in the intention-to-treat population was likely driven by those with *ESR1-*mutant disease (16). Subgroup analysis in patients with *ESR1-*mutant tumors demonstrated that prior antiestrogen and CDK4/6 inhibitor therapy lasting more than 12 months was associated with longer median PFS on elacestrant (8.6 vs. 1.9 months on standard endocrine therapy; HR, 0.41; 95% CI, 0.26–0.63; ref. 17). In this population, elacestrant was associated with superior PFS across several genomic subgroups, including *ESR1 Y537S/N*-mutant tumors (9.0 vs. 1.9 months), *D538G*-mutant tumors (9.0 vs. 1.9 months), and tumors with co-occurring *PIK3CA* mutations (5.5 vs. 1.9 months; ref. 17).

Elacestrant has since been integrated into routine clinical practice for this biomarker-selected patient population. Real-world evidence has emerged as a powerful tool to evaluate the treatment patterns and impact of anticancer agents being deployed in broad, diverse populations as part of routine care (18). Real-world studies leveraging large datasets can explore biomarker associations with patient outcomes. We have previously utilized this approach to examine the impact of *ESR1* mutations on CDK4/6 inhibitor efficacy, which suggested that *ESR1* variants are not associated with resistance to CDK4/6 inhibition (19). The objective of this study was to analyze a large, real-world clinical–genomic database to assess for clinical and molecular factors associated with differences in elacestrant outcomes among patients with *ESR1-*mutant MBC.

## Materials and Methods

### Patients and data source

A cohort of patients was identified for analysis via the GuardantINFORM database, which contains molecular sequencing and treatment information of more than 42,000 breast cancer cases. GuardantINFORM is a large, real-world clinical–genomic dataset with genetic sequencing information from Guardant360 (G360) testing that is linked to administrative claims data. All patients included had G360 sequencing performed in the United States, and this database is generally representative of the demographics and treatment exposures of patients with breast cancer across the United States (Supplementary Table S1). A limitation of this dataset is that race and ethnicity information are not available for analysis.

G360 is a liquid biopsy assay that uses targeted next-generation sequencing of ctDNA to evaluate a panel of genes for insertions, deletions, single-nucleotide variants, amplifications, and select fusion events. The G360 assay utilized during this study assessed 74 to 83 genes, depending on the year and test used. This test has been clinically validated and is commercially available (20, 21).

Data from GuardantINFORM were queried between June 2014 and March 2024, and patient diagnostic and treatment information was extracted via claims records. Claims data for this study were obtained through a commercial aggregator, and unique patient identifiers from source data were hidden using a token-based algorithm to anonymize patient information. For analysis, we included patients who had HR+/HER2− MBC (identified by International Classification of Diseases 9/10 codes), were treated with a line of elacestrant therapy after the drugs’ FDA approval in January 2023, and had an activating *ESR1* mutation detected on ctDNA sequencing within 6 months of elacestrant initiation. Patients without G360 testing within 6 months of elacestrant start and patients without ≥28 days of follow-up after the first elacestrant claim were excluded. Claims records can impose limitations related to missing or incomplete data. Therapy may be stopped because of toxicity or cost rather than disease progression, and administrative claims might incompletely capture prior drug exposures. Patients could lose health insurance or be lost to clinical follow-up, and claims data lack detailed clinical annotation at the individual patient level to discern these differences. The eligibility criteria in this study were utilized to decrease the degree of missing data and minimize the risk of bias.

### Clinical outcomes and lines of therapy

To assess clinical outcomes on elacestrant therapy, treatment regimens from claims data were examined. Accurately defining therapy lines can be a methodologic challenge associated with real-world analyses, and utilizing real-world claims to define lines of anticancer therapy in this context has been previously described (19, 22–26). In this study, the first administration of an anticancer regimen after metastatic cancer diagnosis was defined as the start of the first line of treatment. Agents started within 21 days of each other were considered a combination regimen. Starting a new drug outside of this 21-day window was defined as a subsequent line of treatment. One or more of the drugs in a combination being stopped was not considered a new line of therapy. Between-drug administration within the same line was limited to a 90-day window.

Clinical outcomes were measured via the time-to-treatment-discontinuation (TTD) and time-to-next-treatment (TTNT) as surrogate outcomes for PFS (27), as well as overall survival (OS). The TTD was defined as the start of a line of therapy until treatment claim discontinuation or death, whichever occurred first, and the TTNT was defined as the start of a line of therapy until the start of a subsequent line of therapy or death, whichever occurred first. Patients still on therapy at their last documented activity were censored. OS was defined as the time from starting elacestrant until death, and those without a known death date were censored at last documented activity. Patients that had conflicting claims information for an outcome defining event, for example, multiple different documented dates of death, were excluded from outcomes analysis.

### Genomics

Activating mutations in *ESR1* were defined by genomic variants in the *ESR1* allele that were classified as oncogenic or likely oncogenic in OncoKB (OncoKB.org, RRID: SCR_014782; ref. 28). PI3K pathway–altered status was determined based on the presence of a detectable *PIK3CA*, protein kinase B 1 (*AKT1*), or *PTEN* alteration via baseline G360 testing. For this analysis, we defined patients as having PI3K pathway–altered disease by the presence of one or more variants in *PIK3CA/AKT1/PTEN* that are both oncogenic and therapeutically actionable using a PI3K or AKT inhibitor approved for advanced HR+ breast cancer (29–31). Specifically, we included *AKT1 E17K* mutations, loss-of-function *PTEN* mutations [based on their OncoKB definition (28)], and the following *PIK3CA* alterations as pathway-defining events: “R88Q,” “N345K,” “C420R,” “E542K,” “E545A,” “E545D,” “E545Q,” “E545k,” “E545G,” “Q546E,” “Q546K,” “Q546R,” “Q546P,” “M1043V,” “M10431,” “H1047Y,” “H1047R,” “H1047L,” and “G1049R.” All other PI3K pathway tumor variants were defined as PI3K pathway–unaltered disease.

### Statistical methods

Median TTD, TTNT, and OS were estimated using the Kaplan–Meier method, and nonadjusted survival curves were generated with 95% CIs. A Cox regression model was used to estimate HRs and 95% CIs after adjusting for patient age (continuous variable), sex (female vs. male), Elixhauser comorbidity index (categorical variable), year of G360 testing (2014–2016, 2017–2019, or 2020–2024, chosen based on changes to the gene coverage of the test over time), and line of therapy elacestrant was given (third-line or less. vs. fourth-line or more). All analyses were conducted using R studio version 4.4.1 (RRID: SCR_001905).

### Ethics approval

GuardantINFORM is a completely deidentified database that adheres to article 164.514(a)-(n)1ii of the US Health Insurance Portability and Accountability Act. Informed written consent from individual patients was waived by the Advarra Institutional Review Board, as the collection and analysis of retrospectively obtained, statistically deidentified clinical data were determined to pose minimal risk to participants. This research was conducted in accordance with the ethical guidelines of the Declaration of Helsinki.

## Results

### Demographics

Within our clinical–genomic dataset, a total of 8,730 patients with HR+/HER2− MBC had an *ESR1*-activating mutation detected via ctDNA sequencing. Linking our genomic sequencing results with elacestrant claims data, we identified a cohort of 756 patients who received two or more elacestrant prescriptions after FDA approval and had next-generation sequencing performed within 6 months prior to treatment initiation (Supplementary Fig. S1). The mean age of this group was 63 years, and nearly all patients were female, consistent with the general demographics of breast cancer appreciated in national cancer registries (Table 1; ref. 32). Expectedly, a majority of patients had osseous involvement by their metastatic disease, whereas liver, lung, and then brain metastases were the other most common sites of distant involvement; approximately a quarter of patients had bone-only metastasis.

**Table 1.** Study cohort baseline characteristics.

Characteristic	*N*/Mean	%/SD
Total *N*	756	
Age (years)	​	​
18–49	100	13%
50–64	322	43%
65+	334	44%
Mean (SD)	63	11.8
Sex	​	​
Female	749	99%
Most frequent metastasis sites	​	​
Bone	554	73%
Brain	71	9%
Liver	237	31%
Lung	115	15%
Number of prior lines of metastatic therapy	​	​
0	68	9%
1	138	18%
2	152	20%
3	113	15%
4+	285	38%
Prior therapy exposures in the metastatic setting	​	​
Aromatase inhibitor	594	79%
Fulvestrant	393	52%
CDK4/6 inhibitor	573	76%
Ribociclib	436	58%
Palbociclib	92	12%
Abemaciclib	163	22%
One prior line of CDK4/6 inhibitor	330	44%
Two or more prior lines of CDK4/6 inhibitor	243	32%
Chemotherapy	285	38%
Alpelisib	77	10%
Trastuzumab deruxtecan	58	8%
Sacituzumab govitecan	28	4%
Time from metastatic diagnosis to elacestrant initiation (months)	​	​
0–12	169	22%
12–24	106	14%
24–36	104	14%
36–48	89	12%
48–60	65	9%
60+	155	21%
Mean (SD)	35.4	26.9

Patients in this real-world cohort were treated with a range of therapies prior to elacestrant, with approximately half of the population receiving elacestrant as a third-line or earlier therapy option in the metastatic setting. More than half of the cohort (52%) had previously been treated with fulvestrant, and most patients (76%) had progressed on a prior line of CDK4/6 inhibitor therapy. Nearly 40% of the cohort had received 1 or more lines of chemotherapy before elacestrant initiation, and 12% of patients were previously exposed to an antibody–drug conjugate (ADC; trastuzumab deruxtecan or sacituzumab govitecan).

### Clinical outcomes

**Figure 1.**
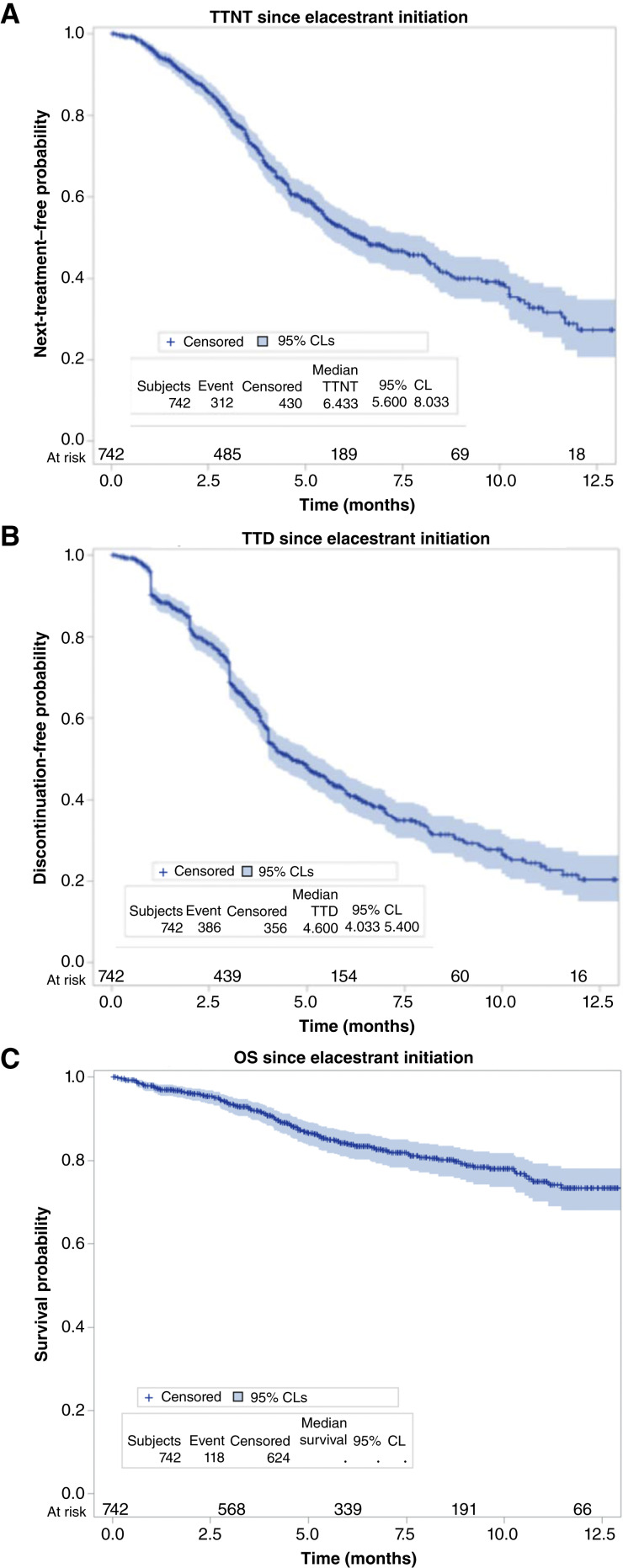
Clinical outcomes in patients treated with elacestrant. **A** depicts the TTNT since elacestrant initiation in this cohort of patients with MBC and *ESR1* mutation detected within 6 months prior to therapy start. **B** displays the TTD in this cohort, and **C** displays the OS. Vertical hash marks denote a censored patient event. CL, confidence limit.

A total of 742 patients were eligible for outcomes analysis on elacestrant, with 14 patients excluded because of conflicting data in claims reports. In this substantially pretreated population, we found that the overall median TTNT on elacestrant was 6.4 months (95% CI, 5.6–8.0) and the median TTD was 4.6 months (95% CI, 4.0–5.4; Fig. 1A–C). The median OS was not reached with 118 events observed. Patients with activating *ESR1* mutations detected more than 6 months before starting elacestrant were excluded from the primary analysis (*n* = 238, with *n* = 235 evaluable for outcomes). This subgroup was analyzed separately for internal validation of the dataset, with similar median TTNT and TTD observed (Supplementary Fig. S2).

**Figure 2.**
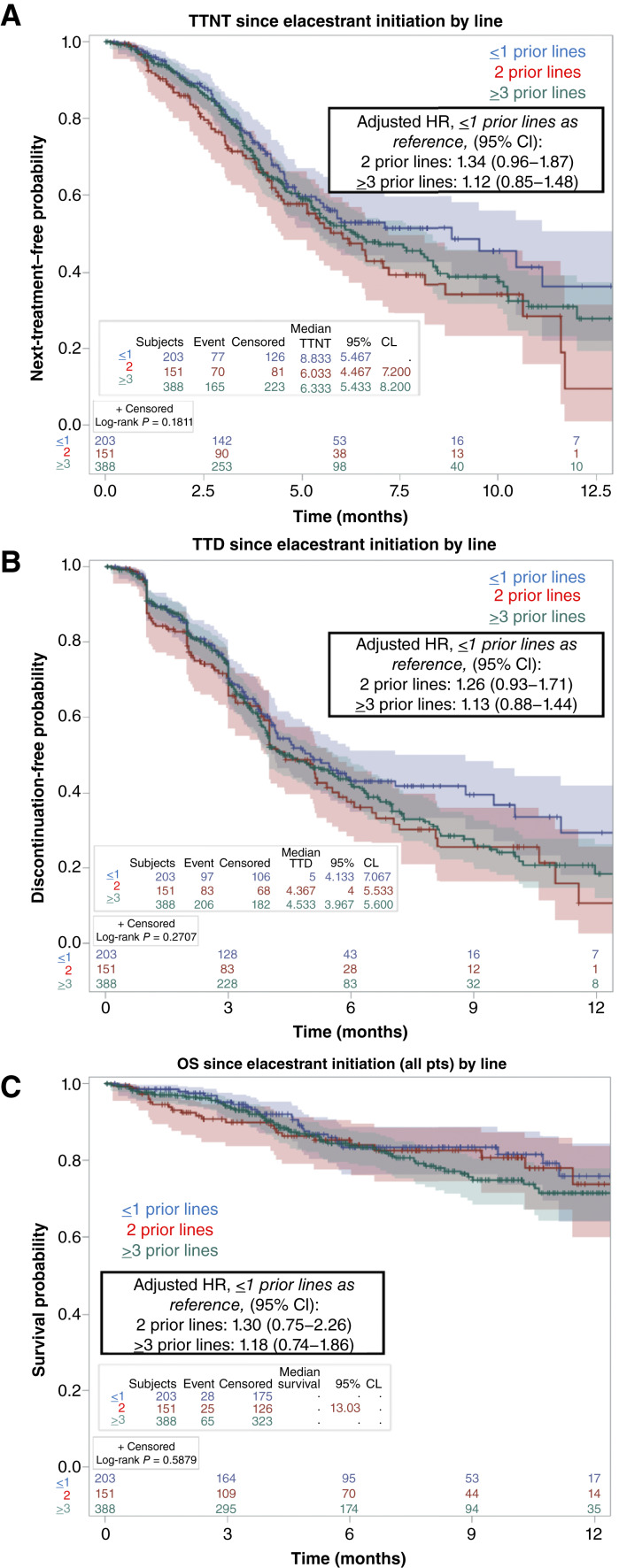
Patient outcomes on elacestrant by prior lines of therapy. TTNT (**A**), TTD (**B**), and OS (**C**) on elacestrant are depicted comparing differences in outcomes by the number of prior lines of metastatic therapy. Patients who received elacestrant after one or fewer prior lines are displayed in blue, after two prior lines are displayed in red, and after three or more prior lines are displayed in green. Adjusted HR and corresponding 95% CI are displayed comparing patients with two prior lines and three or more prior lines with those with one or fewer prior lines of metastatic therapy before elacestrant initiation. Vertical hash marks denote a censored patient event. CL, confidence limit; pts, patients.

We assessed elacestrant outcomes by the number of prior lines of therapy patients had received. In those with one or fewer prior lines of metastatic therapy, the median TTNT on elacestrant was 8.8 months (95% CI, 5.5–not reached) and the median TTD was 5.0 months (95% CI, 4.1–7.1; *n* = 203; Fig. 2A). The median TTNT and TTD in the third-line setting were 6.0 (95% CI, 4.5–7.2) and 4.4 months (95% CI, 4.0–5.5), respectively (*n* = 151; Fig. 2B). Comparing patients with one or fewer prior lines of therapy against those with two, or three or more prior lines, we observed no statistically significant difference in elacestrant treatment duration (Fig. 2A–C).

**Figure 3.**
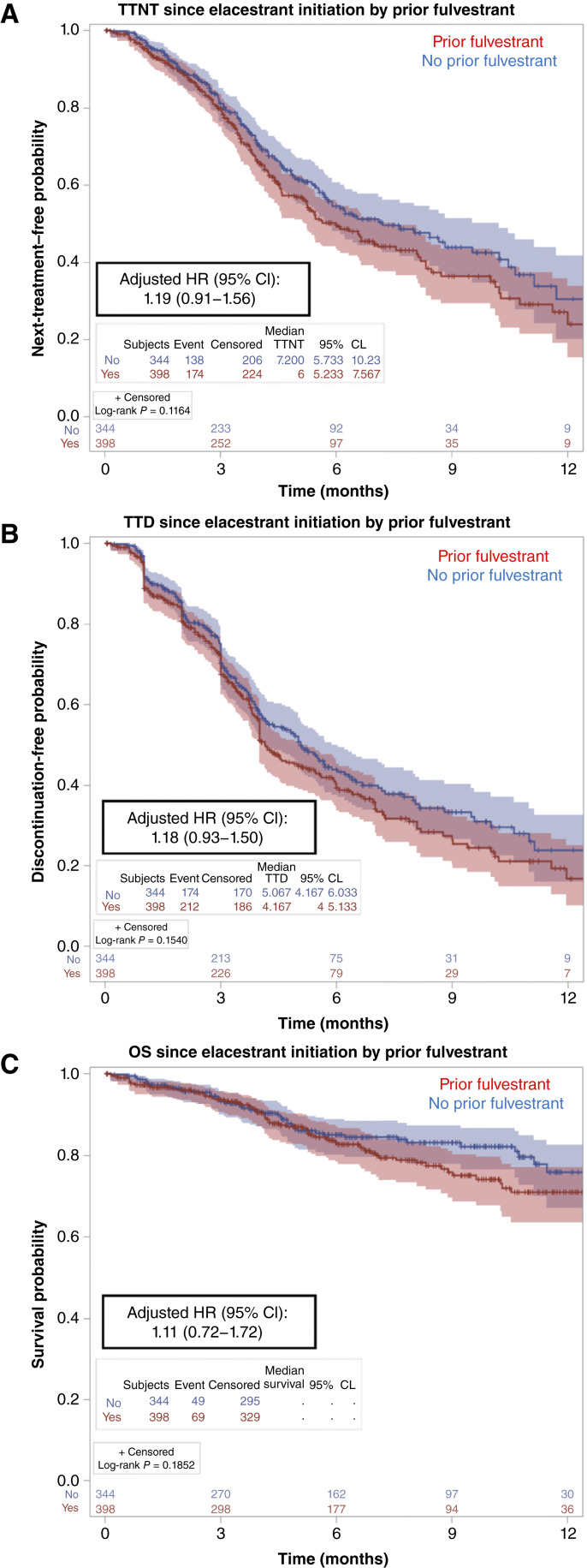
Elacestrant therapy duration and OS by prior fulvestrant exposure. Depicted are clinical outcomes on elacestrant therapy comparing patients with versus those without previous treatment with fulvestrant. Patients treated with fulvestrant before elacestrant are displayed in red, and those without prior fulvestrant are displayed in blue. **A** depicts the TTNT, **B** depicts the TTD, and **C** depicts the OS. The adjusted HR and corresponding 95% CI comparing the two groups are displayed. Vertical hash marks denote a censored patient event. CL, confidence limit.

To evaluate for prior therapy exposures that may correlate with elacestrant efficacy, we compared clinical outcomes in patients previously treated with fulvestrant (*n* = 398) against patients without prior fulvestrant exposure (*n* = 344). Our data suggest that patients who have progressed on prior fulvestrant trended toward shorter TTNT (median 6.0 vs. 7.2 months; adjusted HR, 1.19; 95% CI, 0.91–1.56) and TTD (median 4.2 vs. 5.1 months; adjusted HR, 1.18; 95% CI, 0.93–1.50) on elacestrant; however, statistical significance was not reached (Fig. 3A and B). Furthermore, there was no clear association in terms of OS (adjusted HR, 1.11; 95% CI, 0.72–1.72; Fig. 3C). In patients with prior CDK4/6 inhibitor exposure for metastatic disease (*n* = 561), the median TTNT and TTD on elacestrant were 6.1 and 4.3 months, respectively, compared with 10.3 and 5.3 months in those without prior CDK4/6 blockade (*n* = 181) (TTNT adjusted HR = 1.52, 95% CI, 1.12–2.07; TTD adjusted HR = 1.10, 95% CI, 0.85–1.43; Supplementary Fig. S3). Brain metastases, present in fewer than 10% of patients (*n* = 71), were associated with shorter median TTNT (4.5 vs. 6.6 months; adjusted HR, 1.47; 95% CI, 1.04–2.09), TTD (3.7 vs. 4.9 months; adjusted HR, 1.33; 95% CI, 0.96–1.84), and OS (adjusted HR, 2.22; 95% CI, 1.37–3.60) compared with patients without brain involvement (*n* = 671; *survival curves not shown*)*.*

### Genomic alterations

**Figure 4.**
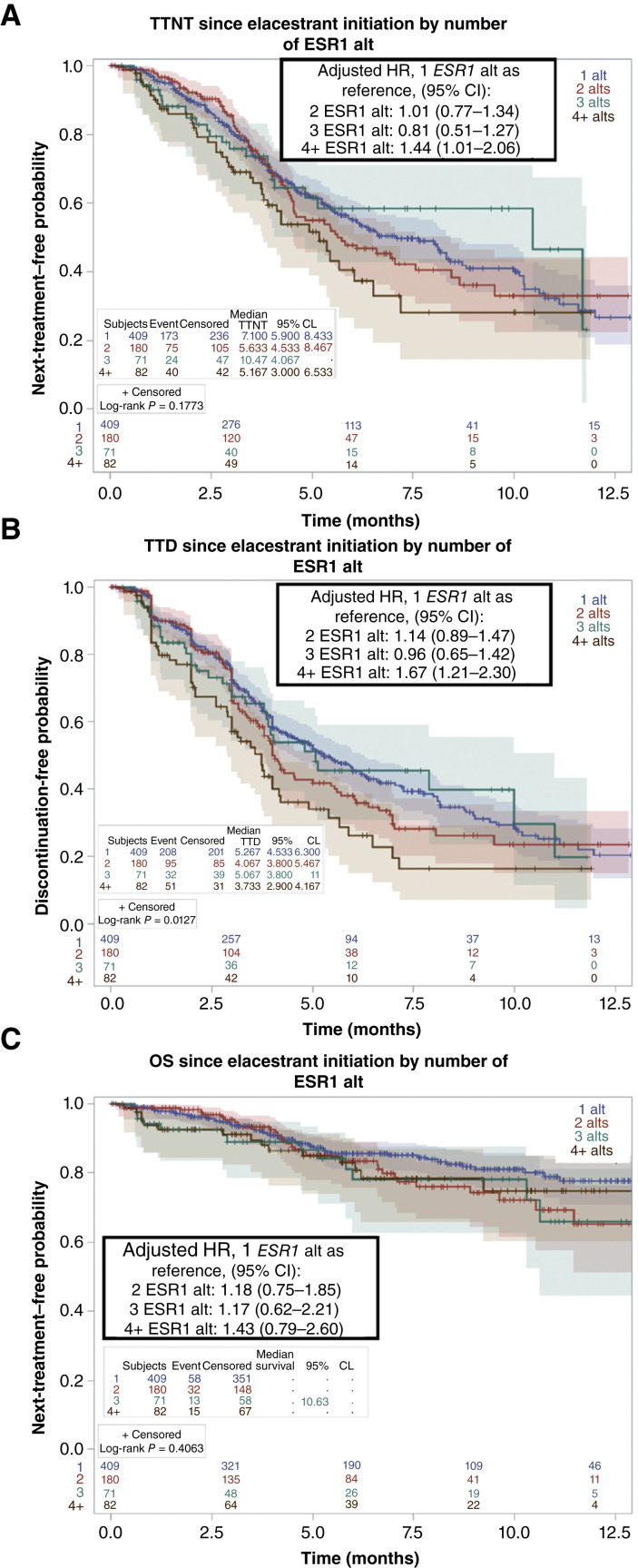
Differences in elacestrant efficacy in patients with polyclonal versus single *ESR1*-mutant tumors. Among this cohort of patients with a baseline *ESR1* mutation detected within 6 months of elacestrant start, clinical outcomes in patients with two, three, or four or more detectable *ESR1* alterations were compared with patients with one *ESR1* mutation. **A** depicts the TTNT on elacestrant, **B** depicts the TTD, and **C** depicts the OS comparing those with two *ESR1* mutations in red, three in green, and four or more in brown against patients with a single *ESR1* mutation in blue. The adjusted HR and corresponding 95% CI are displayed. Vertical hash marks denote a censored patient event. *ESR1* alt denotes *ESR1* alteration. alt, alteration; CL, confidence limit; pts, patients.

All patients in this real-world cohort had at least 1 *ESR1* mutation detected within 6 months prior to elacestrant initiation. The most common *ESR1* alterations detected were *D538G* (69%), *Y537S* (51%), *Y537N* (19%), and *E380Q* (10%; Supplementary Table S2). Nearly half of the patients demonstrated some degree of polyclonality, with >1 *ESR1* mutation detected via ctDNA sequencing at baseline. In patients with evaluable outcomes, we compared elacestrant duration in tumors with a high burden of *ESR1*-mutant polyclonality (≥4 *ESR1* alterations; *n* = 82) versus those with a single *ESR1* mutation (*n* = 409). We observed that the presence of four or more *ESR1*-mutant clones was associated with worse TTNT (median 5.2 vs. 7.1 months; adjusted HR, 1.44; 95% CI, 1.01–2.06) and TTD (median 3.7 vs. 5.3 months; adjusted HR, 1.67; 95% CI, 1.21–2.30; Fig. 4A and B). No difference was seen between patients with tumors expressing two or three *ESR1* mutations compared with a single *ESR1* mutation (Fig. 4A and B), suggesting that higher degrees of polyclonality (≥4 concurrent ESR1 alterations, 11% of patients) could be associated with diminished response to elacestrant. The OS was similar between all groups (Fig. 4C).

We examined outcomes by specific *ESR1*-mutant alleles to determine whether certain variants correlated with elacestrant response. The population with an *ESR1 Y537S* alteration (*n* = 376), compared with those without this variant (*n* = 366), had very similar TTNT (adjusted HR, 0.99; 95% CI, 0.79–1.25) and TTD (adjusted HR, 1.05; 95% CI, 0.85–1.30) on elacestrant (Supplementary Fig. S4). No differences in TTNT or TTD were seen when comparing those exclusively with an *ESR1 Y537S* variant (*n* = 183) against those with an *ESR1 D538G* variant (*n* = 226; *survival curves not shown*).

**Figure 5.**
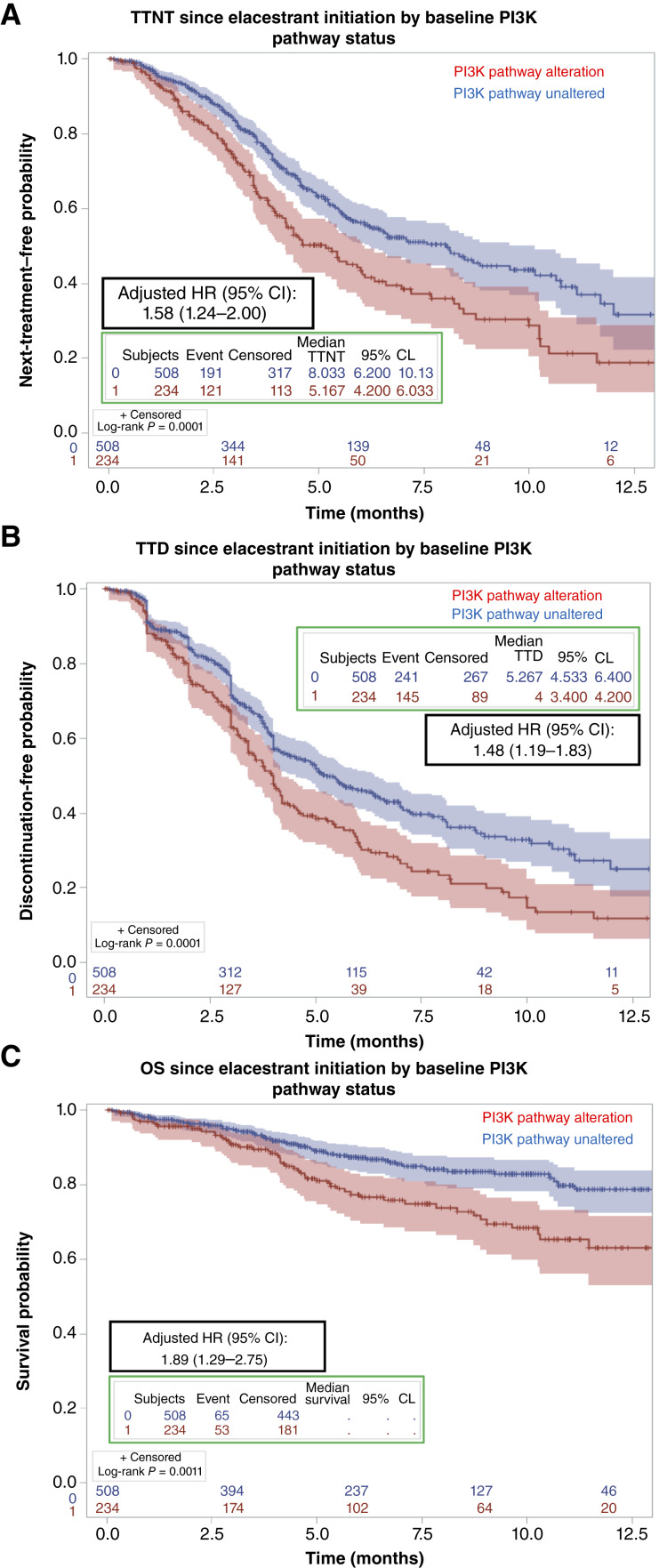
PI3K pathway alterations are associated with differences in clinical outcomes on elacestrant. Patients with PI3K pathway–altered tumors were defined by the presence of an oncogenic *PIK3CA*, *AKT1*, and/or *PTEN* alteration detected within 6 months prior to elacestrant exposure. Clinical outcomes in patients with PI3K pathway–altered disease (in red) were compared with those without a PI3K pathway alteration (in blue) via the TTNT (depicted in **A**), TTD (depicted in **B**), and OS (depicted in **C**). The adjusted HR and corresponding 95% CI are displayed. Vertical hash marks denote a censored patient event CL, confidence limit.

To explore the impact of other genomic alterations on clinical response to elacestrant, we analyzed patient outcomes by PI3K pathway status, which is a clinically relevant and targetable signaling pathway in MBC (29–31). Patients were considered to have PI3K pathway–altered disease if an oncogenic and actionable *PIK3CA*, *AKT1*, or *PTEN* alteration was detected via baseline ctDNA within 6 months prior to drug start. Most patients in this subgroup had a detectable *PIK3CA* mutation (*n* = 197), whereas *AKT1* (*n* = 30) and *PTEN* (*n* = 15) alterations were seen with less frequency, consistent with prior studies in HR+ MBC (2). Among 15 patients with disease harboring loss-of-function *PTEN* alterations, all 15 had single-nucleotide variants detected, and 2 had concurrent deletions in the gene. In patients with PI3K pathway–altered breast cancer (*n* = 234), the median TTNT was 5.2 months (95% CI, 4.2–6.0) and the median TTD was 4.0 months (95% CI, 3.4–4.2; Fig. 5A and B). Compared with those without PI3K pathway–altered disease, the TTNT (median 5.2 vs. 8.0 months; adjusted HR, 1.58; 95% CI, 1.24–2.00), TTD (median 4.0 vs. 5.3 months; adjusted HR, 1.48; 95% CI, 1.19–1.83), and OS were shorter (adjusted HR, 1.89; 95% CI, 1.29–2.75; Fig. 5A–C). In the subgroup of patients with co-occurring PI3K pathway mutations, we compared outcomes in those with prior alpelisib or capivasertib exposure (*n* = 54) against those without (*n* = 184). We observed that the median TTNT (4.5 vs. 5.4 months; adjusted HR, 1.47, 95% CI, 1.05–2.07) and TTD (3.1 vs. 4.1 months; adjusted HR, 1.43, 95% CI, 1.04–1.96) on elacestrant were statistically significantly shorter in patients with prior exposure to alpelisib or capivasertib, though the absolute difference was 1 month or less (*survival curves not shown*).

## Discussion

This clinical–genomic analysis provides robust insight into the real-world deployment of elacestrant for patients with *ESR1-*mutant MBC. We explored outcomes from more than 750 patients treated with elacestrant to evaluate for clinical and molecular factors associated with differences in treatment response, and to our knowledge, this represents the first and largest effort to date examining the use of a next-generation oral ER degrader in routine practice.

A majority of patients in this cohort were more heavily pretreated than patients in the EMERALD study population, including 38% versus 22% of patients with prior chemotherapy exposure, 12% with prior ADC exposure (8% trastuzumab deruxtecan, 4% sacituzumab govitecan), and 52% versus 31% with previous fulvestrant treatment (16). Despite this, TTNT and TTD outcomes in this real-world population compare favorably with the PFS reported in the EMERALD trial (16). In patients with *ESR1*-mutant MBC, the EMERALD study demonstrated a median PFS of 3.8 months on elacestrant monotherapy as second- or third-line treatment and 8.6 months in patients with more than 1 year of benefit on prior endocrine and CDK4/6 inhibitor therapy (16, 17). In our cohort, we observed a median TTNT of 8.8 months and TTD of 5.0 months in the second-line or earlier setting and a median TTNT/TTD of 6.0 and 4.4 months, respectively, in the third-line setting (Fig. 2A and B).

Subgroup analysis of the *ESR1*-mutant EMERALD trial population found that a longer duration of prior antiestrogen plus CDK4/6 inhibitor therapy for ≥12 months was associated with better responses to elacestrant, suggesting this could be a clinical factor that is predictive of endocrine sensitive disease (17). In patients with ≤1 prior lines of therapy, we observed a real-world median TTNT of 8.8 months on elacestrant (Fig. 2A), comparable with the median PFS of 8.6 months reported in this EMERALD subgroup analysis, in which most patients received elacestrant in the second-line setting. Prior CDK4/6 inhibitor duration data were not available in our real-world cohort, limiting our ability to validate whether longer upfront therapy is associated with elacestrant response. However, another real-world study reported that 94% of patients treated with single-agent elacestrant had previously received an antiestrogen and CDK4/6 inhibitor for at least 12 months and found a median real-world PFS of 8.4 months among those with two or fewer prior lines of endocrine therapy (33). Taken together, these data likely reflect real-world practice patterns, in which patients and oncologists may favor an oral SERD monotherapy for *ESR1-*mutant disease that previously responded well to endocrine therapy and CDK4/6 inhibition. In cases of rapid disease progression on an antiestrogen regimen, transitioning to cytotoxic chemotherapy or an ADC may be preferable (2).

Cross-study comparisons should be interpreted with caution due to underlying patient population and methodologic differences; however, the consistent findings across multiple studies, reporting median elacestrant treatment durations of 8 to 9 months in the second- or third-line setting, align with our results. This evidence supports elacestrant monotherapy as an effective therapeutic option for appropriately selected patients with *ESR1-*mutant breast cancer, as reflected in real-world populations treated with this agent for advanced disease. Breast tumors with an *ESR1* mutation, often arising after selective antiestrogen treatment pressure, may depend on constitutive ER pathway signaling and therefore remain sensitive to next-generation oral SERD therapy. We found a trend toward shorter elacestrant responses in patients previously treated with fulvestrant compared with those without prior exposure this agent, and although not statistically significant, this result is not entirely unexpected given that both agents are ER degraders (Fig. 3A and B). We observed that patients without prior CDK4/6 inhibitor exposure in the metastatic setting (a minority of patients with HR+ MBC in routine practice) derived longer benefit on elacestrant, consistent with a population that is less heavily pretreated (Supplementary Fig. S3).

A prior prospective trial testing fulvestrant, with or without palbociclib, for HR+ MBC observed that *ESR1 Y537S* mutations were enriched in fulvestrant-resistant tumors (10). In our study, treatment outcomes on elacestrant were largely consistent across the different *ESR1*-mutant alleles examined. Patients with *ESR1 Y537S* had similar TTNT and TTD when compared with *D538G* or all other *ESR1* variants combined (Supplementary Fig. S4A and S4B). Genomic subgroup analysis from the EMERALD trial similarly showed that elacestrant was equally efficacious for *Y537S/N-* and *D538G-*mutant MBC (17). Patients with ≥4 *ESR1* mutations (observed in approximately 10% of the cohort) had significantly worse TTNT and TTD compared with patients with a single *ESR1* mutation, suggesting a possible relationship between complex *ESR1* mutation polyclonality and diminished response to endocrine therapy (Fig. 4A and B). To our knowledge, this is the first study demonstrating an association between higher degrees of mutant polyclonality and poorer clinical outcomes. Prior studies that dichotomized *ESR1* polyclonality as one versus greater than one mutation did not detect differences in treatment outcomes on fulvestrant (11), potentially due to obscuring a graded effect of increasing polyclonality burden, which warrants deeper investigation in future work.

Tumors with *ESR1* and PI3K pathway co-mutations were associated with a median TTNT of 5.2 months on elacestrant monotherapy, and patients without PI3K pathway alterations experienced longer TTNT/TTD. These results align with the EMERALD subgroup analysis of patients with greater than 12 months on a prior CDK4/6 inhibitor, which showed a median PFS of 5.5 months on elacestrant for breast tumors with dual mutations in *ESR1* and *PIK3CA* (17)*.* Targeted AKT or PI3K inhibition is an emerging therapeutic strategy for many patients with PI3K pathway–altered disease. The AKT inhibitor capivasertib is approved in combination with fulvestrant for *PIK3CA/AKT1/PTEN-*altered advanced breast cancer following results from the CAPItello-291 trial (29). This study demonstrated a median PFS of 7.3 months with capivasertib and fulvestrant doublet therapy in the PI3K pathway–altered population (29) and 5.5 months in patients with prior CDK4/6 inhibitor exposure in the overall population (34). Clinical outcomes in patients with co-occurring *ESR1* and PI3K pathway mutations have not been reported from the CAPItello-291 trial, limiting direct comparisons between elacestrant monotherapy and capivasertib combination therapy in this dual-mutant population. For patients with *PIK3CA-*mutant disease, the PI3K inhibitor alpelisib is approved in combination with fulvestrant based on results from the SOLAR-1 and BYLieve trials (31, 35), and more recently, the PI3K inhibitor inavolisib was approved as part of an upfront triplet regimen with fulvestrant and palbociclib, following the INAVO120 trial for a select group of high-risk patients with recurrence on or within 12 months of completion of adjuvant endocrine therapy (30).

After progression on endocrine therapy and a CDK4/6 inhibitor, concurrent detection of an *ESR1* and PI3K pathway alteration is not infrequent in metastatic breast tumors (approximating 15%–20% of cases; refs. 17, 36, 37), and the optimal therapy selection and treatment sequencing in this setting remains unclear. Our real-world analysis suggests that elacestrant monotherapy retains antitumor activity in these patients with co-occurring, actionable mutations. Clinically, single-agent oral SERD therapy tends to have a more favorable safety profile compared with an AKT or PI3K inhibitor combination, potentially sparing patients from toxicities associated with agents targeting the PI3K pathway, such as rash and hyperglycemia (29, 31). Additional research is ongoing to investigate the efficacy of doublet regimens utilizing a next-generation SERD with a targeted PI3K pathway inhibitor in this population. For example, the ELEVATE trial is a multiarm umbrella study, which includes exploration of the combination of elacestrant plus alpelisib for *PIK3CA-*mutant disease (NCT05563220).

Our study demonstrates strengths and potential limitations of utilizing real-world evidence to inform therapeutic decisions. Real-world data can provide critical insights from routine practice into anticancer therapy use patterns, drug efficacy, and biomarker associations. Large real-world datasets, including GuardantINFORM, provide robust sample sizes for analysis which can far exceed prospective phase III clinical trials. To optimize this biomarker-rich dataset, we restricted our inclusion criteria to evaluate only patients with ctDNA sequencing within 6 months of starting elacestrant. This allowed us to create a study cohort with tumor mutation testing that is more likely to reflect the true genomic alterations present when patients started elacestrant, thus improving the accuracy of our results when examining biomarker associations with treatment outcomes.

Analysis of real-world evidence can be associated with methodologic challenges, and a high frequency of censoring in real-world datasets has the potential to limit the validity of the results. Censoring can result from factors such as data immaturity, loss to follow-up, and transitions in care, all of which reflect the complexity of real-world clinical practice. The observed censoring patterns in this study are consistent with those seen in comparable real-world studies in MBC (19, 38, 39). TTNT and TTD are surrogate outcomes for PFS, which must be carefully considered when interpreting real-world evidence. The inherent differences in TTNT/TTD and PFS measurements can limit direct statistical comparisons with prospective clinical trials; however, this limitation would not affect the internal validity of our findings (40–42).

In this study, we show that elacestrant is associated with a median TTNT exceeding 6 months in a heavily pretreated real-world population with *ESR1*-mutant MBC and a numerically longer TTNT of 8.8 months when used early in the metastatic disease course. These real-world findings serve an important and complementary role in helping to validate the results seen in EMERALD among patients exposed to 1 to 2 prior lines of endocrine therapy, with or without coexisting tumor mutations beyond *ESR1,* and support the routine clinical use of elacestrant in select patients. Our findings in patients with co-occurring PI3K pathway alterations emphasize the importance of personalized therapy selection and suggest that further testing of biomarker-guided regimens holds promise to improve outcomes in patients with concurrent, actionable driver alterations. Many targeted therapies for patients with advanced breast cancer are currently approved, and future biomarker analysis may help elucidate the optimal therapy selection and drug sequencing in the post-CDK4/6 inhibitor treatment landscape. Precision anticancer treatments have potential to improve outcomes and reduce unnecessary toxicity. Future translational investigations should explore additional clinical and molecular factors associated with differences in next-generation SERD efficacy, potentially leading to more refined treatment paradigms, and leveraging real-world evidence is a powerful tool in the analysis and validation of these research efforts.

## Supplementary Material

Supplementary Appendix 1 Supplemental tables 1-2, supplemental figures 1-4

## Data Availability

The dataset generated and analyzed during the current study is not publicly available due to the use of a third-party healthcare claims database and proprietary collation of source data. Researchers interested in replicating our study are encouraged to contact Guardant Health directly via https://guardanthealth.com/precision-oncology/biopharma-solutions/real-world-evidence/ to discuss recapitulating this work or explore specific queries related to the database.
