# Supplementary material for: Clinical and Genomic Factors Associated with Elacestrant Outcomes in ESR1-Mutant Metastatic Breast Cancer
Source: Clin Cancer Res. 2025 Nov 7;32(1):169–78. doi: 10.1158/1078-0432.CCR-25-3033 (PMC12770935; doi:10.1158/1078-0432.CCR-25-3033)
Supplement: Supplementary Appendix 1 — Supplemental tables 1-2, supplemental figures 1-4 [file ccr-25-3033_supplementary_appendix_1_suppsa1.pdf]

**SUPPLEMENTARY APPENDIX**

**TABLE OF CONTENTS**


| | |
|---|---|
| **Table S1:** Representativeness of study participants | 2 |
| **Table S2:** Baseline *ESR1* mutations | 3 |
| **Figure S1:** Patient inclusion flow diagram | 4 |
| **Figure S2:** Elacestrant outcomes in patients with greater than 6 months between G360 testing and treatment start | 5 |
| **Figure S3:** Elacestrant outcomes by prior CDK4/6 inhibitor exposure | 6 |
| **Figure S4:** Elacestrant treatment duration and overall survival by specific *ESR1* mutant alleles | 7 |

| **Supplemental Table 1:** Representativeness of study participants | |
|---|---|
| Cancer type(s) / subtype(s) / stage(s) / condition | Metastatic breast cancer |
| Special considerations related to: | |
| Sex and gender | Breast cancer primarily affects women, with about 99% of cases occurring in females. |
| Age | More than 80% of breast cancers are diagnosed in women aged 50 years or older in the United States.[1] The median age at breast cancer diagnosis is 62 years.[1] |
| Race or ethnic group | Within the United States, breast cancer occurs most frequently in White and Black women, with incidence rates of 133.7 and 127.8 per 100,000, respectively, while Hispanic and Asian/Pacific Islander women have lower rates at 99.2 and 101.3 per 100,000.[1] Mortality is disproportionately higher among Black women (27.6 per 100,000), which is nearly 40% greater than the rate observed in White women (19.7 per 100,000).[1] Black women are more often diagnosed with de novo metastatic disease compared with White women (8% vs. 5%).[1] |
| Geography | Breast cancer incidence and mortality differ across countries, impacted by access to early detection strategies and specific risk factors within populations. |
| Other considerations | Epidemiologic data characterizing metastatic breast cancer in the United States are relatively limited. Statistics from the National Cancer Institute's Surveillance, Epidemiology, and End Results (SEER) program primarily capture patients who present with de novo metastatic disease. However, this represents only a subset of the overall population with advanced breast cancer, as many individuals experience disease recurrence years after treatment for localized disease. Thus, national datasets underestimate the full burden of metastatic breast cancer in the United States. |
| Overall representativeness of this study | The study population had a mean age of 63 years, which is generally aligned with the median age at breast cancer diagnosis in the United States of 62 years. Nearly all participants were female (99%), reflecting the expected sex distribution of breast cancer. Data on patient race and ethnicity were not available in this study, limiting comparison with national demographic patterns associated with this disease. |

**Supplemental Table 2.** Baseline *ESR1* mutations

| **Total N** | 756 | |
|---|---|---|
| **Most frequent *ESR1* alterations** | **N** | **%** |
| D538G | 518 | 69% |
| Y537S | 385 | 51% |
| Y537N | 146 | 19% |
| E380Q | 77 | 10% |
| Y537C | 32 | 4% |
| **Number of *ESR1* alterations** | **N** | **%** |
| 1 | 413 | 55% |
| 2 | 186 | 25% |
| 3 | 73 | 10% |
| 4+ | 84 | 11% |

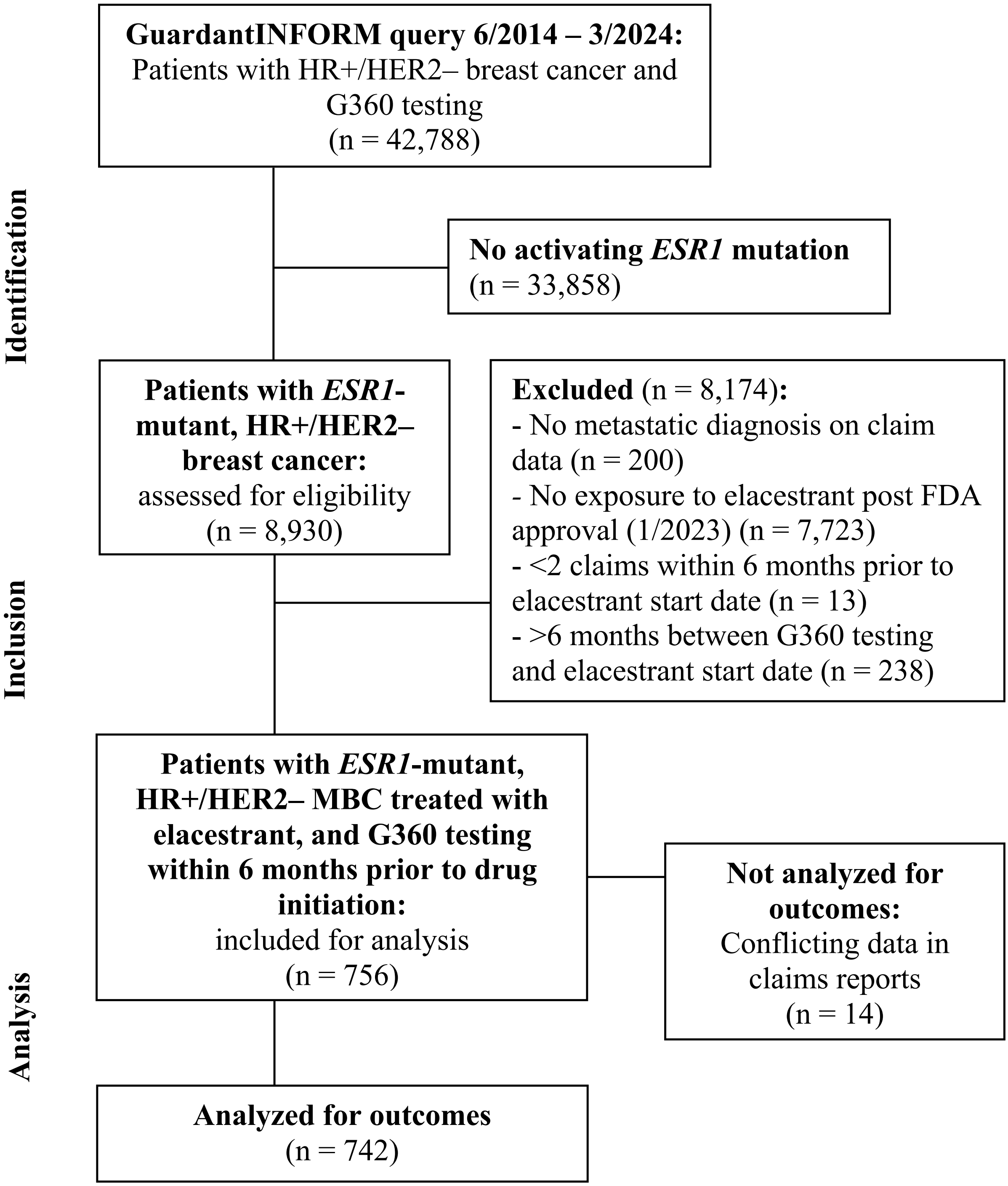


**Supplemental Figure 1. Patient inclusion flow diagram.** This real-world patient cohort was identified using claims data linked with genomic sequencing information. G360, Guardant 360 (a next-generation sequencing assay). HR+, hormone receptor-positive.

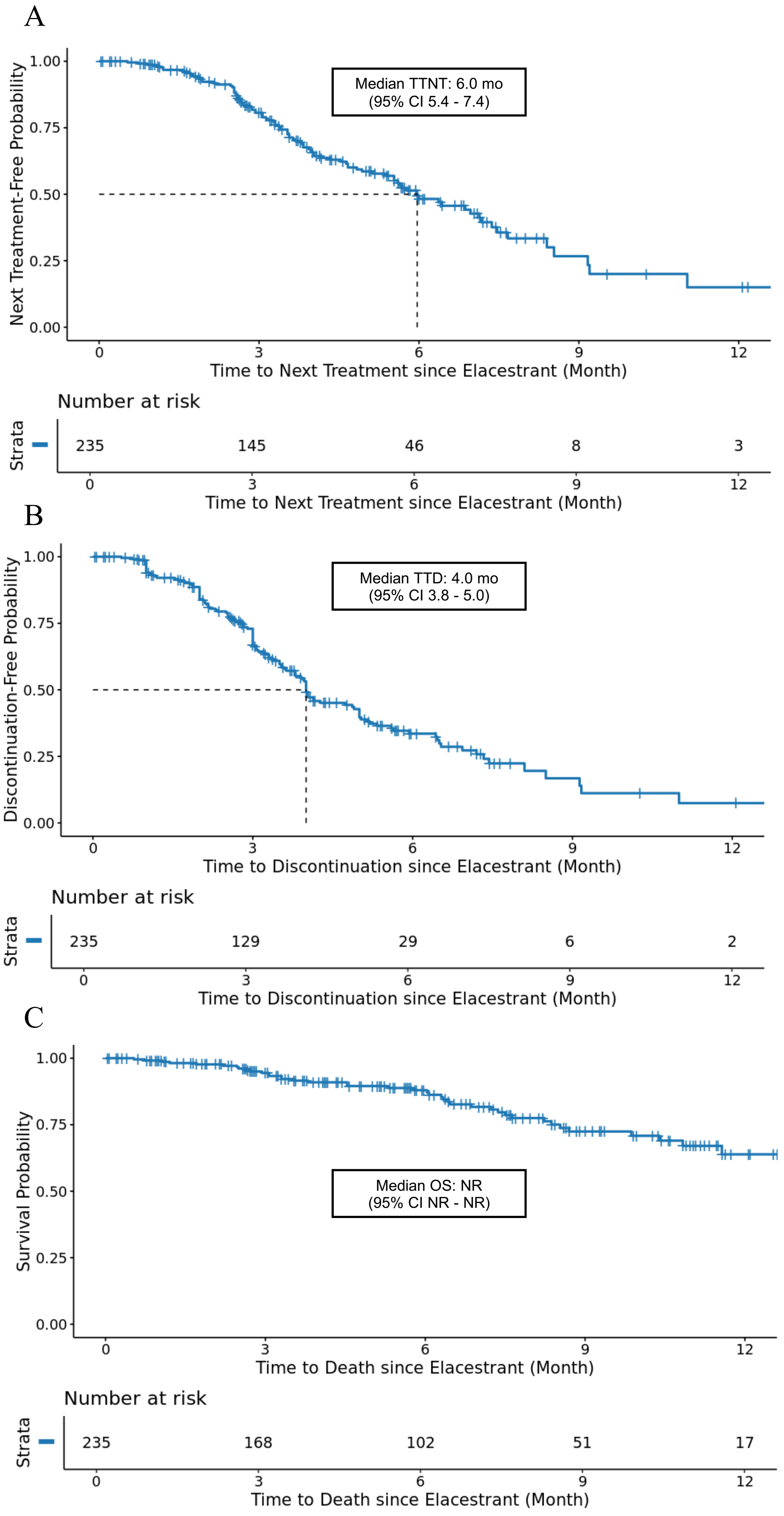


**Supplemental Figure 2. Elacestrant outcomes in patients with greater than 6 months between G360 testing and treatment start.** Patients who received elacestrant but had an activating *ESR1* mutation detected beyond 6 months prior to therapy start were excluded from the primary analysis. For internal validation of the dataset, this population (n=238, and n=235 evaluable for outcomes) were analyzed separately and displayed here. **Panel A** depicts TTNT since elacestrant initiation in this cohort of patients with metastatic breast cancer and *ESR1* mutation detected >6 months prior to treatment start. **Panel B** displays the TTD in this cohort, and **Panel C** displays OS. Vertical hash marks denote a censored patient event. CI, confidence interval; HR, hazard ratio; Mo, months; NR, not reached; OS, overall survival; TTNT, time to next treatment; TTD, time to treatment discontinuation.

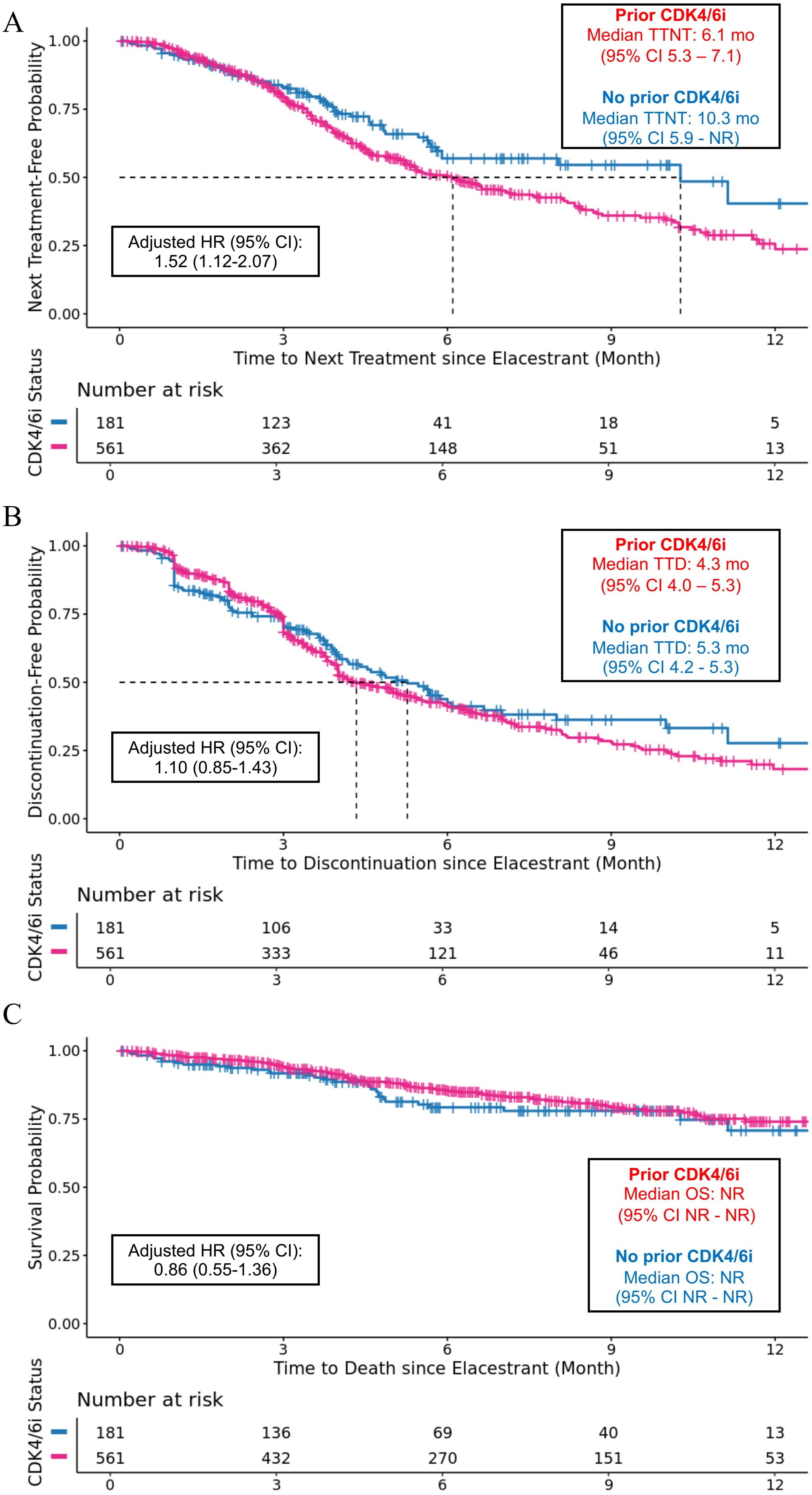


**Supplemental Figure 3: Elacestrant outcomes by prior CDK4/6 inhibitor exposure.** Displayed are clinical outcomes on elacestrant therapy comparing patients with and without prior CDK4/6 inhibitor exposures in the metastatic setting. Patients treated with a line of CDK4/6 inhibitor therapy before elacestrant are displayed in red, and those without prior CDK4/6 blockade are displayed in blue. **Panel A** depicts TTNT, **Panel B** depicts TTD, and **Panel C** depicts OS. The adjusted HR and corresponding 95% CI comparing the two groups are displayed. Vertical hash marks denote a censored patient event. CDK4/6i, CDK4/6 inhibitor; CI, confidence interval; HR, hazard ratio; Mo, months; NR, not reached; OS, overall survival; TTNT, time-to-next-treatment; TTD, time-to-treatment-discontinuation.

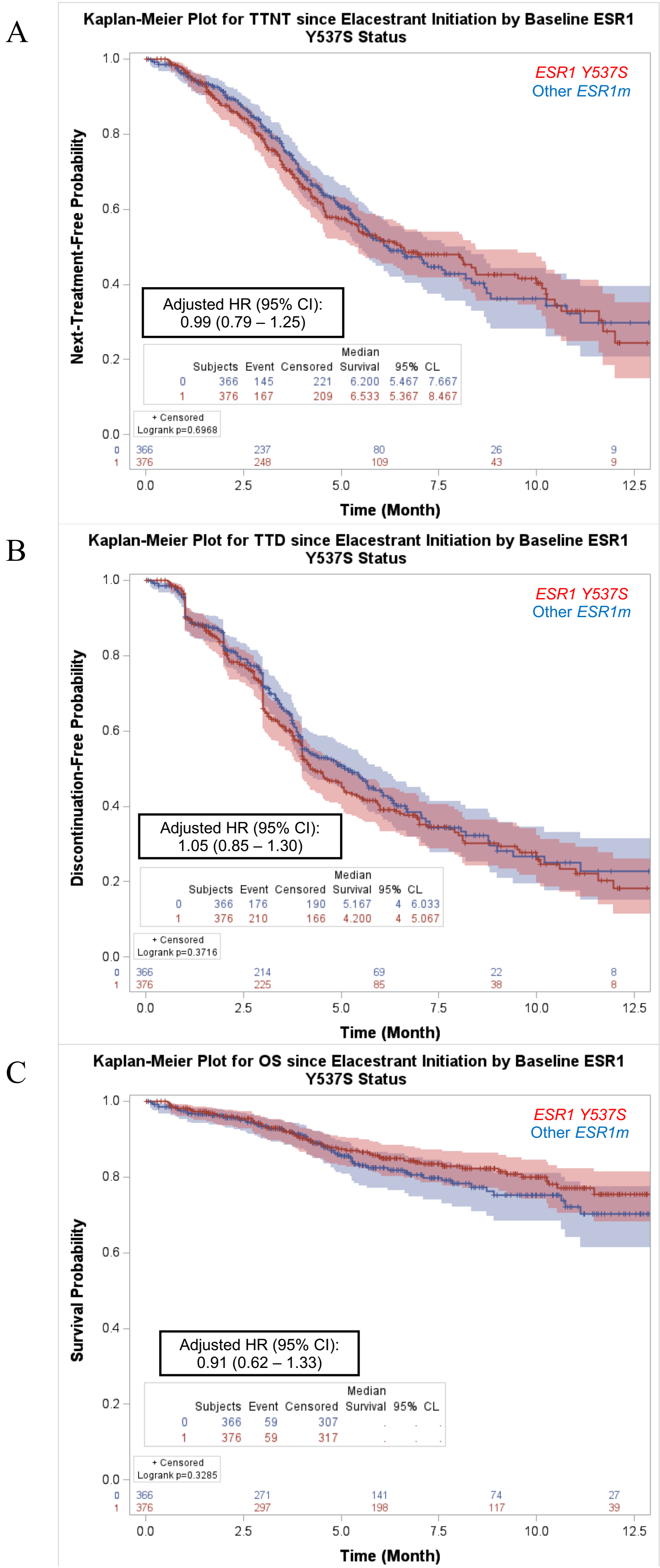


**Supplemental Figure 4. Elacestrant treatment duration and overall survival by specific *ESR1* mutant alleles.** Patient outcomes on elacestrant are depicted comparing those with a baseline *ESR1 Y537S* alteration (in red) to tumors harboring other *ESR1* mutant alleles (in blue). **Panel A** displays TTNT, **Panel B** displays TTD, and **Panel C** displays OS. Adjusted HR and corresponding 95% (CI are displayed. Vertical hash marks denote a censored patient event. *ESR1m* denotes *ERS1* mutation. CI, confidence interval; HR, hazard ratio; OS, overall survival; TTNT, time-to-next-treatment; TTD, time-to-treatment-discontinuation.
